# Initial Arterial pCO_2_ and Its Course in the First Hours of Extracorporeal Cardiopulmonary Resuscitation Show No Association with Recovery of Consciousness in Humans: A Single-Centre Retrospective Study

**DOI:** 10.3390/membranes11030208

**Published:** 2021-03-15

**Authors:** Loes Mandigers, Corstiaan A. den Uil, Jeroen J. H. Bunge, Diederik Gommers, Dinis dos Reis Miranda

**Affiliations:** 1 Department of Intensive Care, Erasmus MC University Medical Center, 3015 GD Rotterdam, The Netherlands; c.denuil@erasmusmc.nl (C.A.d.U.); j.bunge@erasmusmc.nl (J.J.H.B.); d.gommers@erasmusmc.nl (D.G.); 2 Department of Cardiology, Erasmus MC University Medical Center, 3015 GD Rotterdam, The Netherlands

**Keywords:** cardiac arrest, heart arrest, extracorporeal cardiopulmonary resuscitation, extracorporeal membrane oxygenation, carbon dioxide, outcome

## Abstract

Background: Cardiac arrest is a severe condition with high mortality rates, especially in the case of prolonged low-flow durations resulting in severe ischaemia and reperfusion injury. Changes in partial carbon dioxide concentration (pCO_2_) may aggravate this injury. Extracorporeal cardiopulmonary resuscitation (ECPR) shortens the low-flow duration and enables close regulation of pCO_2_. We examined whether pCO_2_ is associated with recovery of consciousness. Methods: We retrospectively analysed ECPR patients ≥ 16 years old treated between 2010 and 2019. We evaluated initial arterial pCO_2_ and the course of pCO_2_ ≤ 6 h after initiation of ECPR. The primary outcome was the rate of recovery of consciousness, defined as Glasgow coma scale motor score of six. Results: Out of 99 ECPR patients, 84 patients were eligible for this study. The mean age was 47 years, 63% were male, 93% had a witnessed arrest, 45% had an out-of-hospital cardiac arrest, and 38% had a recovery of consciousness. Neither initial pCO_2_ (Odds Ratio (OR) 0.93, 95% confidence interval 95% (CI) 0.78–1.08) nor maximum decrease of pCO_2_ (OR 1.03, 95% CI 0.95–1.13) was associated with the recovery of consciousness. Conclusion: Initial arterial pCO_2_ and the course of pCO_2_ in the first six hours after initiation of ECPR were not associated with the recovery of consciousness.

## 1. Introduction

Survival and favourable neurological survival after cardiac arrest are highly influenced by low-flow duration and the associated severity of ischaemia and reperfusion injury [1]. This ischaemia and reperfusion injury is influenced by the level and course of partial oxygen pressure (pO_2_) and partial carbon dioxide pressure (pCO_2_) during and after cardiopulmonary resuscitation (CPR) [2]. During CPR, hypoxemia causes neuron ischaemia and cell death whereas hypercapnia causes cerebrovascular vasodilatation, which may raise intracranial pressure [2]. After regaining circulation, pO_2_ and pCO_2_ will change immediately, which can contribute to reperfusion injury. Especially in the case of hyperoxemia, oxygen-free radicals will be produced causing intracellular oxidation. If hypocapnia occurs, this will result in cerebrovascular vasoconstriction, causing a decreased CBF [2]. Clinically, the importance of regulating pO_2_ during and after conventional CPR has already been proven [3,4,5]. However, research in pCO_2_ values is more limited and the results vary a lot [3,5,6,7].

In an attempt to limit ischaemia and reperfusion injury, extracorporeal cardiopulmonary resuscitation (ECPR) can be used to restore circulation to vital organs as soon as possible. The use of this ECPR enables very fast oxygenation and decarboxylation. However, it is not clear whether these changes in pO_2_ and pCO_2_ should occur rapidly. In patients treated with ECPR, hypoxemia as well as hyperoxemia are associated with lower survival rates [8,9]. The best neurological survival outcomes are seen in patients with normoxia [8]. Despite the possible effects of pCO_2_ in ischaemia and reperfusion, limited studies have been performed on the course of pCO_2_ during and after ECPR. A recent study showed that a large decrease of pCO_2_ after initiation of extracorporeal membrane oxygenation (ECMO) for respiratory failure is associated with neurological complications [10].

We hypothesise that in ECPR, a rapid decrease in pCO_2_ could simulate the occurrence of hypocapnia, leading to cerebral vasoconstriction, which could have a negative impact on neurological outcomes. Therefore, the aim of this study was to investigate the association between pCO_2_ in the first hours after initiation of ECPR and the recovery of consciousness.

## 2. Materials and Methods

We performed a retrospective study at the Erasmus University Medical Centre in Rotterdam, the Netherlands. This hospital has a local database in which all adult patients treated at the emergency department and/or patients of ≥16 years old admitted to the intensive care unit (ICU) for adults, treated with ECMO are registered. The study was conducted in accordance with the Declaration of Helsinki, and the Medical Ethics Committee of the Erasmus University Medical Centre reviewed and approved the study protocol (number MEC-2019-0681). The need for informed consent was waived.

### 2.1. Patients

All patients treated with ECMO who received ECPR in the period 1 January 2010 until 1 January 2020 were selected. After initiation of ECPR, at least three arterial blood gas analyses within the first 6 h had to be known. We excluded all patients with return of spontaneous circulation (ROSC) before ECPR initiation. In our hospital, we consider ECPR in both out of hospital cardiac arrest (OHCA) and in-hospital cardiac arrest (IHCA) patients when the following criteria are met: age ≤ 70 years, witnessed cardiac arrest (last seen < 5 min), good quality of basic life support (BLS) or advanced life support (ALS) leading to an end-tidal carbon dioxide >1.33 kPa, maximum no-flow time of 5 min, a low flow duration of <60 min at the start of ECPR placement, no known terminal illnesses, and no impairment of daily living activities.

### 2.2. ECPR Procedure

ECPR placement was performed by an interventional cardiologist, cardiothoracic surgeon or intensivist, depending on the location where the patient resided. This procedure is mostly performed percutaneously and ultrasound-guided. In the case this is not successful, or if it is performed in the operation room, it is performed surgically. The cannulas are placed in the femoral artery and femoral vein. Every patient receives an antegrade cannula in order to perfuse the leg distally from the cannula placement. Cannulation was started at a minimum CPR duration of 20 min. The decision to contact the ECPR team was made by the attending intensivist. For OHCA patients, we first started with ECPR procedures in patients with massive pulmonary embolism as the cause of arrest. Starting from 1 February 2019, every OHCA patient could be eligible for ECPR.

### 2.3. Measured Variables

We extracted the following variables: patient characteristics (sex, age, and body mass index (BMI)); clinical characteristics (Acute Physiology and Chronic Health Evaluation (APACHE) IV-score, witnessed the arrest, OHCA/IHCA, BLS, direct life support (i.e., BLS or ALS); no-flow duration, low-flow duration, mechanical compression device, primary cardiac rhythm, location of arrest, cause of arrest, laboratory results, and complications); and outcomes (primary outcome: recovery of consciousness and secondary outcomes: ECMO survival, ICU-survival, hospital survival, and cause of death). All known pre-ECMO data were reported according to the Utstein criteria [11].

### 2.4. Statistical Analysis

The distribution of the variables was tested using the Shapiro–Wilk test. Normally distributed continuous variables were reported as mean and standard deviation (SD), and categorical variables as numbers and percentages (%). Non-normally distributed continuous variables were reported as median and interquartile ranges (IQR). To study statistical differences of continuous variables, we used the unpaired T-test for normally distributed variables and the Mann–Whitney U test for non-normally distributed variables. For the categorical variables, we used the Chi^2^ test or the Fisher’s exact test depending on the numbers in each cell.

To examine the possible effect of pCO_2_ on our primary outcome, we performed a binary logistic regression analysis. We included the following pCO_2_ values: initial pCO_2_ value after initiation of EPCR (defined as the first arterial pCO_2_ after starting ECMO flow or the last pCO_2_ within five minutes before starting ECMO flow), course of pCO_2_ (defined as the slope between the first and last pCO_2_ measurement within 6 h), and the interaction between the initial pCO_2_ and the course of pCO_2_. Next, we performed a binary logistic regression analysis including the initial pCO_2_ value after initiation of EPCR, the maximum decrease of pCO_2_ (defined as the maximum percentage of decrease per hour between two measurements within 6 h after initiation of ECPR), and the interaction between the initial pCO_2_ and the maximum decrease of pCO_2_. As sensitivity analyses, we performed binary logistic regression analysis for sustained regain of consciousness at hospital discharge and expected a neurologically favourable outcome at any time after hospital discharge. This expected favourable neurological outcome was determined by reviewing patient charts. A *p*-value < 0.05 was defined as statistically significant and the analyses were performed in R studio, version 3.6.0.

## 3. Results

In our ECMO database, 99 patients underwent ECPR. We excluded patients with two or less arterial blood gas measurements after initiation of ECPR (n = 11), and we excluded patients with ROSC before ECPR initiation (n = 4). A total of 84 patients were included in this study, of which 32 (38%) had a recovery of consciousness at the ICU. Patient characteristics, clinical characteristics, and outcomes are shown in Table 1.

### 3.1. Clinical Characteristics

The mean age of the patients was 47 years (SD 16), and the majority of the patients were male (n = 53, 63%). Almost half of the patients had an OHCA (n = 38, 45%), and in 78 patients (93%) the arrest was witnessed. The median low-flow duration was 51 min (IQR 37–80). This duration did not significantly differ between patients who recovered consciousness versus patients who did not recover consciousness. Most patients had a non-shockable primary cardiac rhythm (n = 57, 69%). We found no significant difference in the primary cardiac rhythm for patients with and without recovery of consciousness. The cause of arrest was primarily pulmonary embolism (36%) followed by acute coronary syndrome (30%).

### 3.2. Laboratory Results

Patients with the recovery of consciousness had higher median pH values (7.07, IQR 6.84–7.21) than patients without the recovery of consciousness (6.90, IQR 6.79–7.00, *p* < 0.01). No differences were seen in initial pO2 values (*p* = 0.10) and initial lactate values (*p* = 0.14) for patients with the recovery of consciousness and patients without the recovery of consciousness. The decrease in lactate values from initiation of ECPR until six hours after initiation of ECPR was significantly higher in patients with the recovery of consciousness (10.38%/h, IQR 12.98–5.06) than in patients without the recovery of consciousness (6.11%/h IQR 11.12–5.48, *p* < 0.05). No significant differences were found in changes of pH and pO_2_ values from initiation of ECPR until six hours after initiation of ECPR.

### 3.3. Outcomes

In total, 32 patients (38%) could be weaned from the ECMO: 28 (33%) of these patients recovered consciousness. Twenty-five patients (30%) survived ICU admission, and 24 patients (29%) survived until hospital discharge. Of those, only one patient (2%) did not recover consciousness. This patient was transferred to another hospital with a Glasgow coma scale motor score of five. In most cases, the cause of death was neurologic (47%, of which 9% brain death and 39% other neurologic causes).

As shown in Table 2, the initial pCO_2_ or pCO_2_ courses were not associated with the recovery of consciousness. In Supplementary Material Figure S1, we included the courses of pCO_2_ in the first six hours after initiation of ECPR for every individual patient. As a sensitivity analysis, we performed the binary logistic regression for patients who had a sustained recovery of consciousness at hospital discharge. These results are shown in Supplementary Material Table S1. Additionally, we determined an expected neurological favourable outcome based on patient charts (classified as cerebral performance category (CPC) score 1–2) at any time from hospital discharge, shown in Supplementary Material Table S2. We also performed the binary logistic regression based on this outcome. As shown in Supplementary Material Table S3, these sensitivity analyses, no significant differences were found.

The values are displayed as odds ratios with 95% confidence intervals (CI). T0: initial values, pCO_2_: partial carbon dioxide concentration. 

## 4. Discussion

In this study, we found that initial pCO_2_ values and the course of pCO_2_ after initiation of ECPR are not associated with the recovery of consciousness. We also found no significant difference for initial pO_2_ values, course of pO_2_ and pH, and initial lactate values. In patients with recovery of consciousness, we found a significantly higher initial pH and a significantly more rapid decrease of lactate than in patients without recovery of consciousness.

Contrary to our hypothesis, recovery of consciousness was not associated with a less rapid decrease of pCO_2_ in the first hours after initiation of ECPR. Based on these findings, a rapid decrease of pCO_2_ until normocapnia might not negatively influence cerebral perfusion. Some studies performed in patients treated with conventional CPR examined the effects of pCO_2_ on survival. Wang et al. [5] evaluated the presence of hypercapnia and hypocapnia in the first 24 h after hospital arrival. They found increased hospital mortality in the case of any hypercapnia or for final hypocapnia [5]. Helmerhorst et al. [3] found only increased hospital mortality in the case of a single measurement of hypocapnia in the first 24 h in patients admitted to the ICU in the Netherlands. In contrast to these two studies, Vaahersalo et al. [7] found a positive association for the duration of hypercapnia within the first 24 h on good neurological outcome. However, these studies did not evaluate the effects of the course of pCO_2_ on the outcome. Ebner et al. [6] did study this course of pCO_2_ in cardiac arrest patients not treated with ECPR. Similar to our results, they have shown no significant association of maximum amplitude in pCO_2_ with poor neurological outcome. Additionally, they also found no significant difference for an area under the curve analysis for the first four pCO_2_ measurements as well as all pCO_2_ measurements and neurological outcomes [6]. In a study by Bemtgen et al. [12], pCO_2_ values were measured several times in the first 24 h after ECPR initiation. They found no significant difference in survival for patients with hypercapnia, hypocapnia, and normocapnia [12].

In addition to our primary outcome, we found three other results in the univariate analyses. First, we found a trend for lower initial pO_2_ values in patients with recovery of consciousness than in patients without recovery of consciousness. This is in line with the recent study by Halter et al. [9] They have shown that ECPR patients with hyperoxemia had a higher odds ratio (OR) for mortality at day 28 (OR 1.89, 95% confidence interval (CI) 1.74–2.07) [9]. These findings suggest that the outcome may be improved by using an oxygen blender with careful titration of the percentage of oxygen for membrane gas flow.

Second, in this study, patients with recovery of consciousness had a slightly higher initial pH than patients without recovery of consciousness. A comparable result was found by Bemtgen et al. [12], who have shown higher survival rates in patients with higher pH values during the first 24 h after initiation of ECPR. However, when compared with the study of Bartos et al. [13], it seems that pH values before ECPR initiation are not associated with survival with favourable neurological outcomes. Despite the possible positive association of higher pH and favourable outcomes, the differences are small (the difference of median pH between patients with and without recovery of consciousness was 0.17). Therefore, it is still not possible to determine at which pH a patient will or will not be eligible for ECPR.

Last, we found a higher lactate decrease in the first six hours after initiation of ECPR in patients who had recovery of consciousness than in patients without recovery of consciousness. This significant difference in lactate clearance is in line with the results found by Mizutani et al. [14]. They found a neurologically favourable survival rate (cerebral performance category 1–2) of 63.1% in patients with high lactate clearance and a neurologically favourable survival rate of 22.2% in patients with low lactate clearance after ECPR.

This study also had some limitations. First, the arterial blood gas sampling was not scheduled, so the measurements were performed by indication decided by the treating physicians. Therefore, the number of samples as well as the time between the samples was varying a lot and could have influenced the outcome of the study. In order to minimize this influence, we divided the samples into time frames. Second, the number of included patients was quite small. Due to this small sample, we were not able to perform a multivariable logistic regression analysis. There were no significant differences between the patient characteristics and cardiac arrest characteristics of the patients with and without recovery of consciousness. However, some of the cardiac arrest characteristics could have influenced the outcome. Third, we included a high rate of patients with non-cardiac causes of arrest. In order to determine if the initial pCO_2_ or low-flow duration in patients with cardiac versus non-cardiac causes of arrest influenced the outcome, we studied these variables and did not find a statistically significant difference. Fourth, in this study we found high rates of BLS in OHCA patients, short no-flow durations, and limited low-flow durations. In the Netherlands, bystander CPR rates, population educated to perform bystander CPR, and use of an automatic external defibrillator (AED) is high and rising every year [15,16]. In our ECPR program, we select patients with an assumed high chance of favourable outcomes (i.e., patients with witnessed arrest, short no-flow times, high quality of CPR, and low-flow durations of <60 min). The selection of ECPR patients, high CPR education, and AED use probably explains the high rates of direct start of BLS and associated short no-flow durations in this study. Last, due to the hypothesis, it could be that the physicians have adjusted the ECPR settings in order to prevent a rapid decrease in pCO_2_. This could result in lower maximum decreases. Therefore, it would be advisable to repeat this study in another patient sample.

We did not find an association between the course of pCO_2_ in the first hours after initiation of ECPR and the recovery of consciousness. Future studies should focus on performing analyses of arterial blood gas values after initiation of ECPR in order to determine the most optimal ECMO settings for neurological favourable outcomes. These studies should be performed in larger samples and with blood gas analyses at set time points.

## 5. Conclusions

Initial arterial pCO_2_ and the course of pCO_2_ in the first six hours after initiation of ECPR were not associated with the recovery of consciousness.

## Figures and Tables

**Table 1:** Characteristics of extracorporeal cardiopulmonary resuscitation (ECPR) patients for patients who did and did not experience a recovery of consciousness.

	Total	Recovery of Consciousness (N = 32)	No Recovery of Consciousness (N = 56)	*p*-Value
Patient number (%)	84	32 (38.1)	52 (61.9)	
Patient characteristics				
Age in years (SD)	46.9 (15.6)	47.1 (15.5)	46.7 (15.7)	0.91
Sex; Male (%)	53 (63.1)	19 (59.4)	34 (65.4)	0.75
BMI (IQR)	26.3 (24.6–29.8)	26.5 (25.2–30.1)	25.8 (24.5–29.4)	0.40
Clinical characteristics				
APACHE IV-score (SD) (Missing N = 36)	112 (31)	110 (36)	113 (27)	0.74
Witnessed arrest (%)	78 (92.9)	31 (96.9)	47 (90.4)	0.40
OHCA (%)	38 (45.2)	13 (40.6)	25 (48.1)	0.66
BLS (%)	37 (44.0)	15 (46.9)	22 (42.3)	0.85
Direct life support (%)	79 (94.0)	30 (93.8)	49 (94.2)	1.00
No-flow in minutes (IQR)	0 (0–0)	0 (0–0)	0 (0–0)	0.25
Total low-flow duration in minutes (IQR) (Missing N = 3)	51.0 (37.0–80.0)	45.0 (30.0–76.5)	58.0 (40.0–84.0)	0.24
Mechanical compression device, e.g., LUCAS (%)	27 (32.1)	6 (18.8)	21 (41.2)	0.06
Primary cardiac rhythm				
Shockable (%)	26 (31.0)	11 (34.4)	15 (29.4)	0.82
Ventricular fibrillation (%)	23 (27.4)	9 (28.0)	14 (28.1)	1.00
Ventricular tachycardia (%)	3 (3.6)	2 (6.3)	1 (2.0)	0.56
Non-shockable (%)	57 (68.7)	21 (65.6)	36 (70.6)	0.82
Pulseless electrical activity (%)	47 (56.0)	20 (62.5)	27 (54.0)	0.60
Asystole (%)	9 (10.7)	1 (3.1)	8 (16.0)	0.08
Location of arrest				
Home (%)	23 (27.4)	8 (25.0)	15 (28.8)	0.90
Public (%)	13 (15.5)	5 (15.6)	8 (15.4)	1.00
ICU (%)	24 (28.6)	9 (28.1)	16 (30.8)	0.99
Ward (%)	10 (11.9)	3 (9.4)	7 (13.5)	0.73
Emergency department (%)	4 (4.8)	1 (3.1)	3 (5.8)	1.00
Operation room (%)	4 (4.8)	3 (9.4)	1 (1.9)	0.15
Catherisation laboratory (%)	3 (3.6)	3 (9.4)	0 (0.0)	0.14
Other (%)	1 (1.2)	0 (0.0)	1 (1.9)	1.00
Cause of arrest				
Acute coronary syndrome (%)	25 (29.8)	12 (37.5)	13 (25.0)	0.33
Pulmonary embolism (%)	30 (35.7)	11 (34.4)	19 (36.5)	1.00
Tamponade (%)	3 (3.6)	2 (6.3)	1 (1.9)	0.55
Hypothermia/drowning (%)	5 (6.0)	1 (3.1)	4 (7.8)	0.64
Post cardiac surgery (%)	2 (2.4)	0 (0.0)	2 (3.8)	0.52
Myocarditis (%)	3 (3.6)	2 (6.3)	1 (1.9)	0.55
Heart failure (%)	3 (3.6)	2 (6.3)	1 (1.9)	0.55
Hypoxemia (%)	2 (2.4)	0 (0.0)	2 (3.8)	0.52
Sepsis (%)	2 (2.4)	1 (3.1)	1 (1.9)	1.00
Other (%)	7 (8.3)	0 (0.0)	7 (12.5)	0.04
Unknown (%)	2 (2.4)	1 (3.1)	1 (1.9)	1.00
Complications				
Bleeding (%)	56 (66.7)	24 (75)	32 (61.5)	0.30
Limb ischaemia (%)	5 (6.0)	2 (6.3)	3 (5.8)	1.00
Cerebrovascular accident (%)	6 (7.1)	4 (12.5)	2 (3.8)	
Cerebral bleeding (%)	5 (6.0)	3 (9.4)	2 (3.8)	0.36
Cerebral infarction (%)	1 (1.2)	1 (3.1)	0 (0.0)	0.38
Infection (%)	28 (33.3)	16 (50.0)	12 (25.0)	0.04
Acute kidney injury (%)	43 (51.2)	21 (65.6)	23 (44.2)	0.09
CRRT (%)	15 (17.9)	6 (18.8)	9 (17.3)	1.00
Tamponade (%)	6 (7.1)	2 (6.3)	4 (7.7)	1.00
Abdominal compartment syndrome (%)	4 (4.8)	1 (3.1)	3 (5.8)	1.00
Laboratory results				
Initial pCO_2_ in kPa (IQR)	7.3 (5.7–9.9)	7.1 (5.3–8.9)	7.7 (6.0–9.9)	0.30
Course of pCO_2_ in %/h (IQR)	−5.22 (−8.69 to −1.99)	−4.09 (−8.38 to −1.30)	−6.28 (−8.69 to −2.08)	0.37
Maximum decrease pCO_2_ in %/hour (IQR)	0.67 (0.38–1.06)	0.58 (0.24–1.06)	0.72 (0.41–0.82)	0.43
Maximum difference pCO_2_ in %/hour (IQR)	−0.52 (−0.87 to 0.39)	−0.30 (−0.88 to 0.08)	−0.59 (−0.86 to 0.71)	0.76
Initial pO_2_ in kPa (IQR)	25.3 (10.8–43.5)	17.4 (9.4–42.5)	32.8 (11.5–47.1)	0.10
Course of pO_2_ in %/hour (IQR)	−6.29 (−11.65 to 9.31)	−4.26 (−12.85 to 12.53)	−7.15 (−11.30 to 5.97)	0.90
Initial pH (IQR)	6.96 (6.80–7.08)	7.07 (6.84–7.21)	6.90 (6.79–7.00)	<0.01
Course of pH in %/hour (SD)	0.68 (0.53)	0.68 (0.48)	0.69 (0.57)	0.98
Initial lactate in mmol/L (SD)	13.7 (5.8)	12.5 (6.0)	14.5 (5.7)	0.14
Course of lactate in %/h (IQR)	−7.44 (−11.89 to −1.33)	−10.38 (−12.98 to −5.06)	−6.11 (−11.12 to −5.48)	<0.05
Outcomes				
ECMO survival (%)	32 (38.1)	28 (87.5)	4 (7.7)	<0.01
ICU-survival (%)	25 (29.8)	24 (75.0)	1 (1.9)	<0.01
Hospital survival (%)	24 (28.6)	23 (71.9)	1 (1.9)	
				
Cause of death	N = 59	N = 9	N = 50	
Brain death (%)	5 (8.5)	0 (0.0)	5 (10.0)	1.00
Neurology (%)	23 (39.0)	4 (44.4)	19 (38.0)	0.73
Cardiac (%)	4 (6.8)	1 (11.1)	3 (6.0)	0.49
Haemorrhagic shock (%)	2 (3.4)	0 (0.0)	2 (4.0)	1.00
Multi-organ disease (%)	14 (23.7)	2 (22.2)	13 (26.0)	1.00
Persisting cardiac arrest (%)	2 (3.4)	0 (0.0)	2 (4.0)	1.00
Other (%)	7 (11.9)	2 (22.2)	5 (10.0)	0.29

Variables were reported as mean and standard deviation (SD), median and interquartile ranges (IQR), and numbers and percentages (%) when appropriate. ECPR: extracorporeal cardiopulmonary resuscitation, BMI: body mass index, APACHE: Acute Physiology and Chronic Health Evaluation, OHCA: out of hospital cardiac arrest, BLS: basic life support, ICU: intensive care unit, CRRT: continuous renal replacement therapy, ECMO: extracorporeal membrane oxygenation, T0: initial time point, Tmax: maximum time value known within 6 h after initiation, pCO_2_: partial carbon dioxide concentration, pO_2_: partial oxygen concentration. Bleeding was defined as every case of bleeding that needed an intervention (e.g., blood transfusion or surgical repair) and tamponade was defined as blood in the pericardium that needed intervention.

**Table 2:** Binary logistic regression analysis of ECPR patients regarding pCO_2_ measurements and the recovery of consciousness.

	(a)	(b)	(c)	(d)	(e)
Initial pCO_2_	0.93 (0.78–1.09)	0.97 (0.79–1.20)	0.92 (0.65–1.30)	0.94 (0.78–1.12)	0.75 (0.52–1.05)
Course of pCO_2_ in first 6 h		1.03 (0.9–1.13)	1.05 (0.92–1.26)		
Interaction initial and course pCO_2_			0.99 (0.97–1.02)		
Maximum decrease of pCO_2_ in first 6 h				1.07 (0.48–2.30)	0.14 (0.01–2.07)
Interaction initial and maximum decrease pCO_2_					1.29 (0.93–1.88)
N	83	83	83	80	80
Nagelkerke R2	0.03	0.04	0.04	0.10	0.13
AIC	113.82	115.22	117.06	111.36	111.02

## Data Availability

The datasets used and analysed during the current study are available from the corresponding author on reasonable request.

## Supplementary Materials

The following are available online at https://www.mdpi.com/2077-0375/11/3/208/s1, Figure S1: Course of arterial pCO_2_ in patients with and without recovery of consciousness, Table S1: Binary logistic regression analysis of ECPR patients regarding pCO_2_ measurements and persisting recovery of consciousness (GCS 6 at hospital discharge), Table S2: CPC scores and expected CPC scores at any time after hospital discharge, Table S3: Binary logistic regression analysis of ECPR patients regarding pCO_2_ measurements and expected favourable neurological outcome.
