# Supplementary material for: Initial Arterial pCO2 and Its Course in the First Hours of Extracorporeal Cardiopulmonary Resuscitation Show No Association with Recovery of Consciousness in Humans: A Single-Centre Retrospective Study
Source: Membranes (Basel). 2021 Mar 15;11(3):208. doi: 10.3390/membranes11030208 (PMC8001427; doi:10.3390/membranes11030208)
Supplement: Supplementary file 1 [file membranes-11-00208-s001.pdf]

membranes

Supplementary Material

Initial Arterial pCO_2 and its Course in the First Hours of Extracorporeal Cardiopulmonary Resuscitation Show No Association with Recovery of Consciousness in Humans: A Single-Centre Retrospective Study

Loes Mandigers [1,*], Corstiaan A. den Uil [1,2], Jeroen J. H. Bunge [1,2] Diederik Gommers [1] and Dinis dos Reis Miranda [1,*]

[1] Department of Intensive Care, Erasmus MC University Medical Center, 3015 GD Rotterdam, The Netherlands; c.denuil@erasmusmc.nl (C.A.d.U.); j.bunge@erasmusmc.nl (J.J.H.B.); d.gommers@erasmusmc.nl (D.G.)

[2] Department of Cardiology, Erasmus MC University Medical Center, 3015 GD Rotterdam, The Netherlands

* Correspondence: l.mandigers@erasmumc.nl (L.M.); and d.dosreismiranda@erasmusmc.nl (D.d.R.M.); Tel. DdRM: +31-010-703-5142

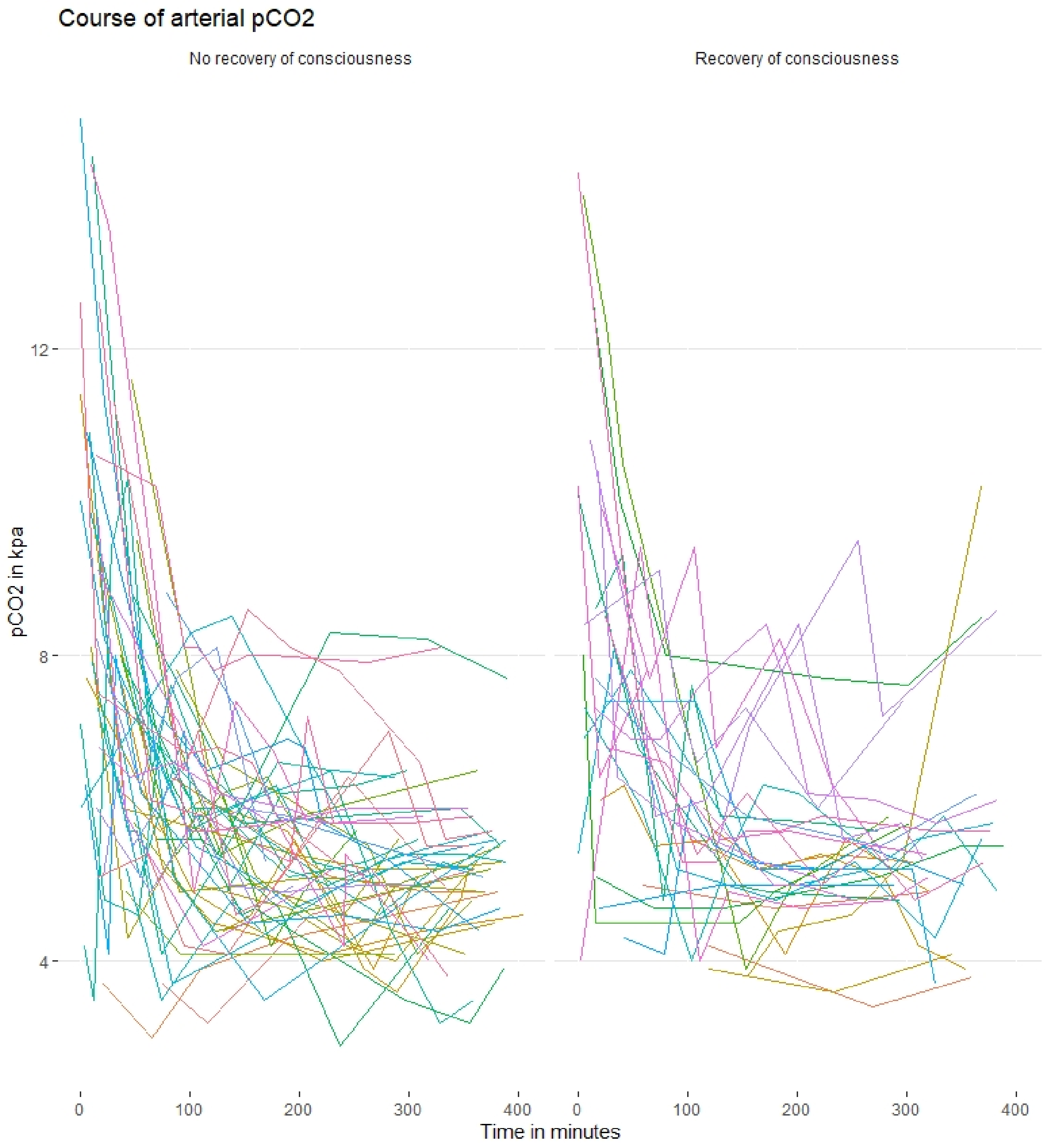

Figure A. Course of arterial pCO_2 in patients with and without recovery of consciousness

Course of the arterial pCO_2 in the first six hours after initiation of extracorporeal cardiopulmonary resuscitation of every individual patient divided in patients with and without recovery of consciousness. Partial carbon dioxide concentration (pCO_2).

Table A. Binary logistic regression analysis of ECPR patients regarding pCO_2 measurements and persisting recovery of consciousness (GCS 6 at hospital discharge).

	(a)	(b)	(c)	(d)	(e)
Initial pCO_2	0.97 (0.81-1.14)	1.04 (0.83; 1.29)	1.11 (0.76; 1.67)	0.99 (0.81; 1.20)	0.87 (0.60; 1.23)
Course of pCO_2 in first 6 hours		68.52 (0.04; 1,434,915)	3.98 (0.00000057; 73,391,710)		
Interaction initial & course pCO_2			1.92 (0.13; 57.72)		
Maximum decrease of pCO_2 in first 6 hours				1.13 (0.47; 2.53)	0.34 (0.02; 5.73)
Interaction initial & maximum decrease pCO_2					1.16 (0.83; 1.67)
N	83	83	83	80	80
Nagelkerke R2	0.01	0.04	0.04	0.11	0.12
AIC	115.20	102.56	104.40	98.00	99.24

The values are displayed as odds ratios with 95% confidence intervals. T0: initial values, pCO_2: partial carbon dioxide concentration.

Table B; CPC scores and expected CPC scores at any time after hospital discharge

**Expected CPC score of 1-2**	N =
CPC 1-2 at CPC questionnaire	6
EQ-5D-5L questionnaire: independent functioning	6
Functioning in home environment	6
Working again	1
**Expected CPC score 3 or unknown outcome**	
Unknown; transferred to other hospital with M6 score	3
CPC 3 at CPC questionnaire (both living at home, one cannot function independently for 24 hours a day and 1 needs care for physical problems)	2

In some patients CPC score was tested by a questionnaire (Mak et al, 20..), in other patients the CPC score was estimated based on information reported in the patient files. CPC = cerebral performance category

Table C. Binary logistic regression analysis of ECPR patients regarding pCO_2 measurements and expected favourable neurological outcome.

	(a)	(b)	(c)	(d)	(e)
Initial pCO_2	0.98 (0.81-1.18)	1.14 (0.89; 1.47)	1.47 (0.94; 2.41)	1.03 (0.83; 1.26)	0.89 (0.60; 1.30)
Course of pCO_2 in first 6 hours		4169.16 (0.80; $3.79*10^9$)	0.05 ($2.78*10^{-10}$; $8.54*10^7$)		
Interaction initial & course pCO_2			13.15 (0.34; 1055.56)		
Maximum decrease of pCO_2 in first 6 hours				1.08 (0.83; 2.54)	0.27 (0.01; 6.08)
Interaction initial & maximum decrease pCO_2					1.18 (0.82; 1.74)
N	83	83	83	80	80
Nagelkerke R2	<0.01	0.07	0.11	0.13	0.15
AIC	90.78	89.15	89.37	85.92	87.09

The values are displayed as odds ratios with 95% confidence intervals. T0: initial values, pCO_2: partial carbon dioxide concentration.
